# Spatially resolved tryptophan–kynurenine niches in HNSCC: immunometabolic microdomains and therapeutic implications

**DOI:** 10.3389/fimmu.2026.1756010

**Published:** 2026-01-28

**Authors:** Wanchen Ning, Shaohong Huang

**Affiliations:** Stomatological Hospital, School of Stomatology, Southern Medical University, Guangzhou, Guangdong, China

**Keywords:** aryl hydrocarbon receptor (AhR), HNSCC, IDO1/TDO2, immunometabolic niches, spatial heterogeneity, tryptophan–kynurenine pathway

## Abstract

Head and neck squamous cell carcinoma (HNSCC) develops within a chronically inflamed tumor microenvironment (TME) where metabolic reprogramming and immune suppression tightly co-evolve. A prominent example is the tryptophan–kynurenine (Trp–Kyn) pathway, initiated by indoleamine 2,3-dioxygenase 1/2 (IDO1/IDO2) and tryptophan 2,3-dioxygenase (TDO2), which converts tryptophan into kynurenine and downstream metabolites that engage stress-response programs and aryl hydrocarbon receptor (AhR) signaling. In HNSCC, Trp–Kyn enzymes are inducible by interferons and oncogenic cues and are distributed across malignant cells as well as cancer-associated fibroblasts, endothelial cells and tumor-associated myeloid populations, generating spatially restricted “Trp-low/Kyn-high” immunometabolic niches. Within these niches, tryptophan starvation and kynurenine-driven signaling converge to suppress effector T-cell expansion, promote regulatory T-cell programs, undermine dendritic-cell priming and reinforce tolerogenic myeloid states, collectively fostering T-cell exhaustion and reduced sensitivity to PD-1/PD-L1 blockade. Emerging bulk, single-cell and spatial multi-omics studies support the idea that pathway activity is compartmentalized rather than uniform, providing a mechanistic rationale for the limited performance of first-generation IDO1 inhibitor strategies in unselected clinical settings. This mini-review synthesizes current evidence on Trp–Kyn microdomains in the HNSCC TME and discusses therapeutic opportunities that move beyond single-enzyme inhibition, including dual IDO1/TDO2 targeting, AhR antagonism and biomarker-guided combination regimens to restore antitumor immunity.

## 1 Introduction

Head and neck squamous cell carcinoma (HNSCC) is one of the most aggressive epithelial malignancies worldwide, accounting for hundreds of thousands of new cases and deaths each year (1–5). Its development is strongly associated with well-established carcinogenic exposures, including tobacco use, excessive alcohol consumption and high-risk human papillomavirus (HPV) infection (6). Although advances in surgery, radiotherapy and platinum-based chemotherapy have improved local control, patients with advanced or recurrent HNSCC still face an unfavorable prognosis, and the overall five-year survival rate rarely exceeds 50% (7–9). Immune checkpoint inhibitors (ICIs) targeting the PD-1/PD-L1 axis have brought clinical benefit to a subset of patients, yet objective response rates remain modest at approximately 15%–30% (10), underscoring the need to better understand the mechanisms of immune resistance within the tumor microenvironment (TME).

Metabolic reprogramming is a hallmark of cancer, supporting tumor growth while reshaping the surrounding microenvironment. Changes in glucose, lipid, and amino acid metabolism provide energy and biosynthetic substrates and can also modulate immune-cell differentiation, trafficking, and effector function (11). In HNSCC, enhanced aerobic glycolysis, increased fatty-acid synthesis, and dysregulated amino acid utilization have been associated with immune escape and reduced therapeutic responsiveness (12). Among amino acid pathways, tryptophan (Trp) metabolism is particularly notable because it directly couples metabolic flux to immune regulation: ~95% of free Trp is degraded through the kynurenine (Kyn) pathway, initiated by the rate-limiting enzymes indoleamine 2,3-dioxygenase 1/2 (IDO1/IDO2) and tryptophan 2,3-dioxygenase (TDO2) (13). This pathway links amino acid metabolism to immune suppression in the TME (14).

Recent evidence indicates that in HNSCC, abnormal activation of the Trp–Kyn pathway is largely driven by inflammatory cytokines, such as interferon-γ, together with tumor-derived factors that induce robust expression of IDO1 and TDO2 in malignant and stromal cells (15, 16). Multi-omics profiling and spatial transcriptomics further show that this metabolic axis is unevenly distributed across tumor nests, stromal compartments and immune cell aggregates, pointing to region-specific immune–metabolic interactions within the tumor microenvironment (17–19). Such intricate cross-talk promotes T-cell exhaustion, extracellular matrix remodeling and resistance to therapy, thereby positioning the Trp–Kyn pathway as a crucial link between metabolic reprogramming and immunosuppression in HNSCC.

Taken together, defining the Trp–Kyn axis and its spatially localized activity is essential for understanding immune escape in HNSCC and for guiding metabolism–immune combination strategies. In this review, we summarize key molecular and immunological features of this pathway in HNSCC and outline emerging therapeutic approaches, aiming to inform biomarker-guided immunotherapy optimization.

## 2 The tryptophan-kynurenine pathway in HNSCC

### 2.1 Overview of the Trp–Kyn pathway

The kynurenine (Kyn) pathway is the major route of tryptophan (Trp) catabolism, responsible for ~95% of Trp degradation under physiological conditions (20). Its first and rate-limiting step is mediated by indoleamine 2,3-dioxygenase 1 (IDO1), IDO2, and tryptophan 2,3-dioxygenase (TDO2), which convert Trp to N-formylkynurenine and subsequently to kynurenine. Kynurenine is further metabolized into bioactive derivatives such as 3-hydroxykynurenine, anthranilic acid, and quinolinic acid, which can modulate redox balance and immune programs, thereby influencing tumor growth, inflammatory signaling, and immune tolerance (16).

In cancer, the Trp–Kyn pathway functions as an immunometabolic checkpoint that integrates nutrient availability with immune regulation. Hyperactive IDO1 or TDO2 in tumor cells and stromal compartments can deplete local Trp, thereby limiting a critical substrate for proliferating effector T cells. Trp scarcity activates the general control nonderepressible 2 (GCN2) kinase–eukaryotic translation initiation factor 2α (eIF2α) stress-response axis in T cells, promoting cell-cycle arrest and functional exhaustion (21). In parallel, accumulation of Kyn and downstream metabolites activates the aryl hydrocarbon receptor (AhR), facilitating regulatory T-cell (Treg) expansion, myeloid-derived suppressor cell (MDSC) recruitment, and upregulation of inhibitory receptors such as PD-1 and CTLA-4. Together, these mechanisms establish an immunosuppressive tumor microenvironment (TME) that enables immune escape and tumor progression (22).

### 2.2 Enzyme expression and regulation in HNSCC

Aberrant activation of the Trp–Kyn axis has been widely reported in HNSCC. Compared with adjacent normal mucosa, HNSCC tissues frequently exhibit elevated expression of IDO1 and TDO2, and higher levels of these enzymes have been associated with advanced clinical stage, lymph node metastasis and unfavorable prognosis (15, 18). Mechanistically, IDO1 is strongly inducible in response to inflammatory cytokines within the tumor microenvironment, particularly interferon-γ (IFN-γ). In representative HNSCC cell lines (e.g., FADU, Detroit-562 and Cal-33), IFN-γ stimulation markedly increases IDO1 expression, consistent with an adaptive feedback program in which antitumor immune pressure promotes local Trp catabolism and immune restraint (15). In parallel, TDO2 expression is also observed in HNSCC and may contribute to sustained Trp-to-Kyn conversion across distinct tumor contexts and cellular compartments, supporting the notion that Trp–Kyn flux can be maintained through complementary enzymatic routes beyond IDO1 alone. By contrast, IDO2 remains less well characterized in HNSCC, and its relative contribution likely depends on tumor subtype and microenvironmental cues.

### 2.3 Spatial heterogeneity within the tumor microenvironment

Recent spatial and single-cell transcriptomic studies highlight pronounced heterogeneity in the distribution of Trp–Kyn enzymes across the HNSCC tumor microenvironment (23–25). IDO1 and TDO2 are detected not only in malignant epithelial cells but also in cancer-associated fibroblasts (CAFs), tumor-associated macrophages (TAMs), and endothelial cells (18, 24, 26). This compartmentalized expression is consistent with localized immunometabolic niches in which Trp depletion and Kyn accumulation occur near infiltrating lymphocytes and may spatially constrain antitumor immune function. Such organization further supports a bidirectional link between immunity and metabolism in HNSCC, whereby stromal and myeloid compartments reinforce Trp catabolism, while immune-derived cytokines (e.g., IFN-γ) sustain enzyme induction and pathway activity. Together, these observations suggest that the Trp–Kyn axis in HNSCC reflects an ecosystem-level adaptation that couples metabolic stress with immune dysfunction across distinct TME microdomains.

### 2.4 Functional biomarkers and their clinical significance

Functional activation of the Trp–Kyn pathway can be assessed using surrogate biomarkers, among which the kynurenine-to-tryptophan (Kyn/Trp) ratio in serum or tumor tissue is most commonly used as an indicator of pathway activity and immunosuppressive potential (27, 28). An elevated Kyn/Trp ratio has been associated with poorer outcomes, increased Treg infiltration and diminished responses to immune checkpoint therapy (21, 29). In addition, transcriptome-based analyses in HNSCC have suggested that higher expression of Trp metabolism–related gene programs correlates with weakened cytotoxic T-cell features and reduced overall survival (21, 30). Together, these biomarkers support the clinical relevance of Trp–Kyn activation and motivate further efforts to integrate metabolic readouts with immune profiling for improved patient stratification.

## 3 Immunological effects of the tryptophan-kynurenine pathway in HNSCC

**Figure 1:**
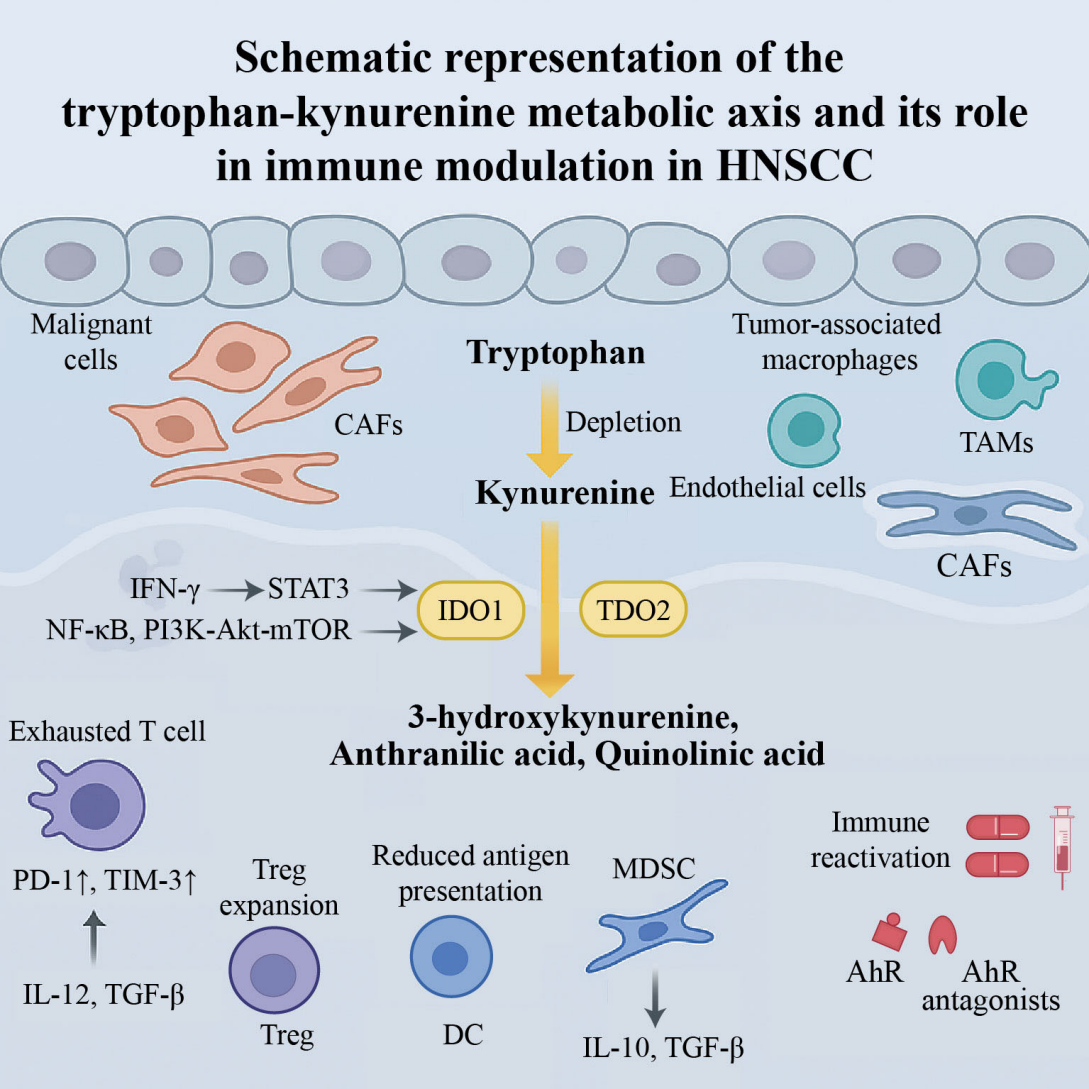
Metabolic–immune interactions of the Trp–Kyn pathway in head and neck squamous cell carcinoma (HNSCC). Activation of IDO1 and TDO2 depletes tryptophan and increases kynurenine, leading to T-cell exhaustion, regulatory T-cell expansion, dendritic cell dysfunction, and MDSC recruitment. Combined inhibition of IDO1/TDO2 and AhR, together with checkpoint blockade, may restore anti-tumor immunity.

The immunometabolic regulatory network orchestrated by the Trp–Kyn pathway in HNSCC is summarized in **Figure 1**. Activation of the Trp–Kyn pathway within the tumor microenvironment (TME) exerts broad effects on both innate and adaptive immunity (14, 31, 32). In head and neck squamous cell carcinoma (HNSCC), dysregulated tryptophan metabolism can reprogram immune cell states, promote immune tolerance and weaken effective antitumor responses (15, 21, 33). Although mechanistic studies specifically dissecting HNSCC remain relatively limited, available evidence in HNSCC supports an association between activation of the Trp–Kyn pathway and an immunosuppressive tumor microenvironment, while mechanistic insights from other solid tumors provide complementary context for how this pathway may contribute to immune dysfunction and reduced responsiveness to immunotherapy.

### 3.1 Tryptophan depletion and T cell dysfunction

One of the earliest and most well-established immunological consequences of Trp–Kyn activation is local tryptophan depletion (34, 35). Effector T cells are particularly sensitive to amino acid scarcity because clonal expansion, protein synthesis and cytokine production require sufficient tryptophan availability. Reduced extracellular tryptophan activates the general control nonderepressible 2 (GCN2) stress-response pathway in T cells, leading to eIF2α phosphorylation, translational restraint and cell-cycle arrest. In the HNSCC TME, tumor cells and stromal compartments with high IDO1/TDO2 activity can generate “tryptophan-low” microdomains that restrict CD8^+^ T-cell infiltration and compromise cytotoxic effector function (17, 18). These microdomains are frequently associated with an exhausted T-cell phenotype, including increased expression of inhibitory receptors such as PD-1 and TIM-3 and diminished IFN-γ production (36). Thus, metabolic starvation and immune checkpoint signaling can operate in parallel to reinforce T-cell dysfunction and limit the depth and durability of immunotherapy responses.

### 3.2 Kynurenine accumulation and aromatic hydrocarbon receptor signaling

Kynurenine (Kyn) and downstream metabolites provide direct immunomodulatory signals beyond tryptophan depletion. Kyn acts as an endogenous ligand of the aryl hydrocarbon receptor (AhR), a transcription factor that controls gene programs involved in immune differentiation and tolerance (37). Upon ligand binding, AhR translocates to the nucleus and induces transcriptional programs that favor immunosuppressive phenotypes (21). In HNSCC, activation of the IDO/TDO–Trp–AhR axis has been linked to regulatory T-cell expansion, recruitment of myeloid-derived suppressor cells, and polarization of tumor-associated macrophages toward immunoregulatory states. This axis may also potentiate immune checkpoint pathways by increasing PD-1 on dysfunctional CD8^+^ T cells and PD-L1 on tumor and myeloid compartments, thereby reinforcing local immune tolerance and contributing to resistance to immune checkpoint inhibitors (ICIs) (38).

### 3.3 Dendritic-cell inhibition and antigen-presentation dysfunction

Dendritic cells (DCs) are essential for initiating antitumor immunity through antigen processing, presentation and priming of naïve T cells. Activation of the Trp–Kyn pathway can disrupt this process by impairing DC maturation and reducing expression of co-stimulatory molecules required for productive T-cell activation (39, 40). IDO1-expressing DCs often acquire a tolerogenic phenotype characterized by increased IL-10 and TGF-β production and reduced IL-12 secretion (41, 42). This cytokine milieu favors Treg differentiation and suppresses effector T-cell activation (43). In HNSCC, tumor-associated DCs located near IDO1^+^ stromal or epithelial compartments have been reported to exhibit reduced antigen-presenting capacity, supporting the concept that metabolic cross-talk can blunt initiation of adaptive immunity (17, 44, 45). Over time, inadequate antigen presentation and ineffective priming contribute to immune-dysfunctional states, and a subset of HNSCC tumors may exhibit “cold” or poorly inflamed immunophenotypes, which are typically associated with lower response rates to checkpoint blockade (46, 47).

### 3.4 Cross-talk with extracellular matrix remodeling and myeloid populations

The immunosuppressive impact of the Trp–Kyn pathway is not confined to lymphocytes. In HNSCC, cancer-associated fibroblasts (CAFs) can express IDO1 and secrete immunomodulatory cytokines, thereby amplifying local Kyn production and reinforcing suppressive niches (18, 48, 49). Tumor-associated macrophages can also engage tryptophan catabolism and, together with prostaglandins and TGF-β signaling, promote immunosuppression while supporting extracellular matrix (ECM) remodeling and tumor invasion (49–51). In parallel, MDSCs can sense Kyn-associated cues and activate immunosuppressive programs (e.g., arginase 1 and inducible nitric oxide synthase, iNOS) via AhR-dependent signaling, further inhibiting T-cell function and integrating into the Trp–Kyn-mediated suppressive network (52–54). Collectively, these multi-layer feedback loops illustrate how metabolic regulation, stromal remodeling and myeloid reprogramming cooperate to sustain immune suppression in the HNSCC microenvironment.

### 3.5 Overall impact on tumor immunity

Taken together, Trp–Kyn metabolism establishes a multilayer immunosuppressive architecture in HNSCC. Through coordinated effects of tryptophan depletion, kynurenine-driven AhR signaling, suppression of antigen presentation and stromal–myeloid reinforcement, this pathway simultaneously weakens cytotoxic immunity, expands regulatory immune populations and constrains immune priming. The resulting TME is characterized by immune dysfunction and metabolic restriction, which can undermine the efficacy of immunotherapy. A clearer understanding of these interconnected mechanisms is therefore critical for designing strategies that disrupt metabolism–immune cross-talk and restore effective antitumor immunity in head and neck cancer.

## 4 Therapeutic targeting of the Trp–Kyn metabolic pathway in HNSCC

Because Trp–Kyn metabolism is a key immunoregulatory axis, it has emerged as a promising therapeutic target in HNSCC. Translational efforts have largely focused on inhibiting the rate-limiting enzymes IDO1 and TDO2 and/or disrupting downstream aryl hydrocarbon receptor (AhR) signaling (31). Although early clinical studies—especially enzyme-directed monotherapies—did not deliver the anticipated benefit, increasing recognition of pathway redundancy, spatial compartmentalization, and intertumoral heterogeneity has shifted attention toward mechanism-guided combination strategies, reviving interest in Trp–Kyn–centered immunometabolic interventions.

### 4.1 IDO1 inhibitors: early prospects and clinical limitations

Indoleamine 2,3-dioxygenase 1 (IDO1) inhibitors were among the first “metabolic immunotherapies” to enter clinical development. In preclinical models, agents such as epacadostat, navoximod and linrodostat demonstrated immune-stimulatory potential, in which IDO1 blockade could alleviate immunosuppressive pressure, restore T-cell proliferation and enhance responses to immune checkpoint blockade (31, 32, 55). However, clinical translation proved challenging. In melanoma, the phase III ECHO-301/KEYNOTE-252 trial reported no survival advantage for epacadostat plus pembrolizumab compared with pembrolizumab alone, prompting a broad reassessment of IDO1 inhibition as a stand-alone strategy and of trial design principles for this axis (56). In HNSCC, early experiences with IDO1 inhibitors have likewise shown limited efficacy, potentially reflecting incomplete suppression of Trp–Kyn flux and/or compensatory activation of parallel enzymes such as TDO2 (57). Importantly, IDO1 expression in HNSCC is often inducible and spatially heterogeneous rather than constitutively high, implying that “one-size-fits-all” dosing and unselected enrollment may fail to achieve adequate target coverage in the relevant microdomains (23, 58). Rather than representing a definitive failure of the pathway, these outcomes emphasize the biological complexity of Trp–Kyn regulation and support clinically rational approaches that incorporate biomarker-based patient selection, pharmacodynamic monitoring and multi-node pathway targeting.

### 4.2 TDO2 inhibition and dual-target strategy

Tryptophan 2,3-dioxygenase (TDO2) can function as a complementary—and in some contexts compensatory—Trp-catabolizing enzyme alongside IDO1. Because TDO2 expression can be prominent in tumor cells (and physiologically in the liver), Trp-to-Kyn conversion may persist even when IDO1 is pharmacologically inhibited, enabling metabolic bypass and sustained immunosuppressive signaling (32, 59, 60). Preclinical studies suggest that dual inhibition of IDO1 and TDO2 more effectively reduces pathway output and can yield stronger immunologic restoration than single-agent blockade (61–63). Accordingly, newer therapeutic concepts include dual inhibitors and combination regimens designed to concurrently target IDO1 and TDO2, and in some designs, additional downstream nodes (e.g., kynurenine aminotransferases, KATs) to more comprehensively suppress Kyn production and prevent metabolic escape (64). Because Trp–Kyn metabolism converges on shared downstream effectors—most notably AhR signaling—dual targeting of enzymatic production and receptor-mediated transcriptional programs is also conceptually appealing, with the aim of interrupting both the metabolic source and the immunosuppressive signaling output of the pathway.

### 4.3 AhR blocking and immune metabolic reprogramming

As a key mediator of kynurenine signaling, AhR has emerged as an alternative (and potentially complementary) therapeutic target within the Trp–Kyn axis (21). Pharmacologic AhR antagonism may restore effector T-cell function, limit regulatory T-cell differentiation and attenuate MDSC-driven immune suppression, thereby counteracting multiple downstream immunosuppressive consequences without relying solely on upstream enzyme inhibition (53, 65). In addition, growing evidence indicates that AhR blockade may increase tumor sensitivity to immune checkpoint inhibitors and reshape transcriptional programs linked to immune and metabolic states within the tumor microenvironment, supporting its role as a candidate node for combination-based immunometabolic reprogramming.

## 5 Discussion

Over the past decade, the Trp–Kyn axis has emerged as a pivotal driver of immunosuppression and treatment resistance across multiple malignancies, including HNSCC (14, 31, 66). This pathway exemplifies how tumor metabolism extends beyond supporting tumor growth to actively reshaping immune surveillance. In HNSCC, the inducible activation of indoleamine 2,3-dioxygenase (IDO1), tryptophan 2,3-dioxygenase (TDO2), and the downstream kynurenine metabolites provides a biochemical link between chronic inflammation, metabolic adaptation and immune tolerance (18). By coupling local tryptophan depletion with accumulation of immunoregulatory metabolites, the Trp–Kyn axis imposes multilayered constraints on antitumor immunity, affecting T cells, dendritic cells, macrophages and stromal fibroblasts within the tumor microenvironment.

A key conceptual advance is the recognition of Trp–Kyn metabolism as an active immune-regulatory circuit rather than a passive by-product pathway. Spatial and single-cell analyses indicate that IDO1/TDO2 expression is highly heterogeneous across tumor nests and stromal compartments, generating microdomain-specific immunosuppression (18, 23, 24).This compartmentalization offers a plausible explanation for the inconsistent clinical performance of single-enzyme–targeted approaches: partial or spatially mismatched pathway blockade may leave metabolite production and downstream signaling intact in critical niches. In this light, negative results with late-stage IDO1 inhibitor trials should be viewed less as evidence against the pathway and more as an indication of redundancy, context dependence and dynamic regulation within the tumor ecosystem.

Translating mechanistic insights into therapeutic benefit requires addressing several challenges. First, enzymatic redundancy across IDO1, TDO2 and tryptophan aminotransferases argues for multi-node inhibition strategies or rational combinations that prevent metabolic bypass. Second, the spatiotemporal variability of enzyme induction underscores the need for biomarker-guided patient selection and pharmacodynamic monitoring; integrated markers such as tumor IDO1/TDO2 expression, the kynurenine/tryptophan (Kyn/Trp) ratio, and transcriptional features consistent with aryl hydrocarbon receptor (AhR) activation may better capture functional pathway activity than any single measurement alone (50, 67). Third, the interplay between systemic metabolism, microbiota-derived tryptophan metabolites and local immune programs remains incompletely defined, yet may critically influence therapeutic responsiveness and should be prioritized in future translational studies.

Looking forward, integrating metabolomics with single-cell and spatial multi-omics can resolve Trp–Kyn activity at high resolution and reveal context-specific vulnerabilities missed by bulk profiling, enabling biomarker-guided combination regimens that align metabolic intervention with immunomodulation. Beyond enzyme inhibition, emerging directions such as AhR antagonism, dietary or microbiota-oriented metabolic remodeling, and nanocarrier-enabled delivery to enhance intratumoral coverage may further advance precision immunometabolic therapy. Overall, the Trp–Kyn pathway in HNSCC reflects immune–metabolic crosstalk and represents a tractable therapeutic axis; better definition of its compartmentalized biology and clinical correlates may help shift it from a marker of immune resistance to a component of metabolism-informed immunotherapy strategies for head and neck cancer.
